# A Comparison Study of the Effect on IBS-D Rats among Ginger-Partitioned Moxibustion, Mild Moxibustion, and Laser Moxibustion

**DOI:** 10.1155/2021/4296216

**Published:** 2021-11-17

**Authors:** Chao Sun, Xiaofeng Yang, Sirui Xie, Ziqin Zhou, Guoliang Yu, Shangsheng Feng, Jingyu Zhao, Jiangtao Wu, Changchun Ji

**Affiliations:** ^1^Key Laboratory of Thermo-Fluid Science and Engineering of MOE, Xi'an Jiaotong University, Xi'an 710049, China; ^2^Department of Pathogenic Microbiology and Immunology, School of Basic Medical Sciences, Xi'an Jiaotong University, Xi'an 710061, China; ^3^Department of Acupuncture and Moxibustion, Shaanxi Traditional Chinese Medicine Hospital, Xi'an 710003, China; ^4^School of Life Science and Technology, Xi'an Jiaotong University, Xi'an 710049, China; ^5^Department of Acupuncture and Moxibustion, Xi'an Traditional Chinese Medicine Hospital, Xi'an 710021, China

## Abstract

**Background:**

Diarrhea-predominant irritable bowel syndrome (IBS-D) is a functional gastrointestinal disorder that severely affects patients' life. Moxibustion is believed to be an effective way to treat IBS-D. However, the therapeutic effects and the underlying mechanisms in symptom management of IBS-D by different moxibustion therapies remain unclear.

**Methods:**

IBS-D model rats were divided into groups and treated with ginger-partitioned moxibustion (GPM), mild moxibustion (MM), and laser moxibustion (LM) at a temperature of 43°C, respectively. The temperature curves of acupoints were recorded during interventions. The therapeutic effects were evaluated on the basis of general condition, stool, and hematoxylin-eosin staining of the colon tissue. Moreover, the expression of transient receptor potential vanilloid 1 (TRPV1) receptors in both acupoint tissue and colon tissue was analyzed by immunohistochemistry.

**Results:**

After moxibustion treatment, the symptoms were improved. The expression of TRPV1 was increased in acupoint tissue and decreased in colon tissue. GPM and MM showed a more significant influence on IBS-D rats compared with LM. The temperature profile of GPM and MM was wave-like, while LM had an almost stable temperature curve.

**Conclusion:**

GPM, MM, and LM could improve the symptoms in IBS-D rats. Moxibustion might activate TRPV1 channels in the acupoint tissue and induce acupoint functions, which in turn inhibit the pathological activation state of the colon's TRPV1, followed by improvements in abdominal pain and diarrheal symptoms. LM with stable temperature might lead to the desensitization of TRPV1 receptors and the tolerance of acupoint. GPM and MM provided dynamic and repetitive thermal stimulations that perhaps induced acupoint sensitization to increase efficacy. Therefore, dynamic and repetitive thermal stimulation is recommended in the application of moxibustion.

## 1. Introduction

Irritable bowel syndrome (IBS) is a chronic functional gastrointestinal disorder characterized by altered bowel habits, abdominal pain, and bloating, with a global incidence as high as 23% [1, 2]. Diarrhea-predominant (IBS-D) is reported as the most common subtype of IBS (28%–46%), with recurrent abdominal pain and diarrhea as the main manifestations [3, 4]. IBS-D is characterized by prolonged treatment and easy relapse and is often accompanied by mental and psychological disorders such as anxiety and depression that will severely affect the quality of patients' lives and consume medical resources. According to the theory of Traditional Chinese Medicine, IBS-D patients have multiple spleen and stomach problems, loss of health and vitality, diarrhea, spleen and kidney issues, and loss of kidney yang. Moxibustion, as one treatment of Traditional Chinese Medicine, is carried out by applying thermal stimulation on specific acupoints, which is believed to prevent and treat IBS-D [5].

With the continuous promotion and innovation of moxibustion therapies, there are more types of moxibustion therapy for clinical treatment of IBS-D. Numerous studies have shown the positive effects of moxibustion therapies for the treatment of IBS-D [6, 7]. Ma et al. [8] observed that herb-partitioned moxibustion greatly alleviated the symptoms of patients with IBS-D, and this appears to be a promising and efficacious treatment for IBS-D. Tong et al. performed mild moxibustion intervention on IBS-D models rats for one week and found that mild moxibustion improved the symptoms of diarrhea significantly [9]. Shi et al. [10] found that both electroacupuncture and moxibustion were effective in treatment of IBS patients. However, the intervention effect of different moxibustion therapies on IBS-D is still unclear, and there has been no consistent standard so far for the choice of moxibustion therapy for the treatment of IBS-D, which hampers the clinical application and promotion of moxibustion intervention in IBS-D.

The intensity of thermal stimulation is the premise for the desired therapeutic effect of moxibustion [11]. As *Lingshu Cijiezhenxie* says, “the fire is energized, the blood is right.” Different intensities of thermal stimulation are related to different therapeutic effects in the treatment of IBS-D [12]. Temperature is a good quantitative form of the intensity of thermal stimulation [13–16]. Local moxibustion temperatures were closely related to the reaction of local receptors, which induced the thermal function of moxibustion [17]. Transient receptor potential vanilloid 1 (TRPV1), which can be activated by noxious thermal stimulation (>43°C) [18], is believed to be a key target in producing the therapeutic effect of moxibustion [19]. However, little attention has been paid to the temperature and its relationship with TRPV1 for the treatment of IBS-D in existing research. In addition, we must pay attention to not only the temperature value reached during moxibustion, but also the process of temperature variation, that is, the temperature characteristics of moxibustion.

In this study, we selected commonly used ginger-partitioned moxibustion, mild moxibustion, and laser moxibustion as thermal stimulation methods and IBS-D as the disease model. We applied moxibustion treatment in IBS-D rats and controlled the temperature of rat skin at approximately 43°C during moxibustion intervention. From the perspective of thermophysics, we investigated the therapeutic effect and molecular biological mechanisms of the three moxibustion therapies in IBS-D rats and explored the relationship of temperature characteristics and therapeutic effects.

## 2. Materials and Methods

### 2.1. Experimental Animals

Forty male Sprague Dawley rats weighing 150 g ± 20 g were supplied by the Laboratory Animal Center of Xi'an Jiaotong University. Each rat was housed in an individual cage. The room temperature and relative humidity were maintained at 20°C∼25°C and 50%∼70%, respectively. All experimental procedures were performed on the basis of the guidelines provided by the National Institute of Health for the Care and Use of Laboratory Animals, and the experimental protocol was approved by the Animal Ethics Committee of Shaanxi Traditional Chinese Medicine Hospital.

### 2.2. IBS-D Modeling and Identification

After three days of adaptive feeding, all rats without adverse reactions were randomly divided into normal group (*n* = 8) and IBS-D modeling group (*n* = 32). IBS-D was induced by intracolonic instillation of 1 mL glacial acetic acid (40 mL/L) at 8 cm proximal to the anus for 30 s. Then, 1 ml phosphate buffered saline (PBS, 0.01 mol/L) was injected to flush the colon [20]. In addition, the restraint stress stimulus was performed by restraining the rat's front shoulders, upper limbs, and chest using wide transparent tape to restrict it from scratching its head and face, lasting 2 h/day for one week [21].

To verify the modified IBS-D model and mental state, fur appearance and activity of rats were monitored. The 24 h food and water intakes were recorded. Weight increases of rats before and after modeling were calculated. Moreover, stools of rats were analyzed by the diarrhea index and Bristol stool scale.

Each rat in the normal group and the modeling group was put into a cage with a sparse grid at the bottom, and a tray was placed under the cage. A piece of filter paper the same size as the tray was put into it. Six hours later, all rats were put back into the feeding cage. The stools contaminated with filter paper were considered as loose stools, otherwise they were dry stools, and the loose stool rate was defined as the percentage of the number of loose stools to the total number of stools. Loose stools were graded by the measured contamination diameters according to Table 1, and then all loose stool grades were summed and divided by the total number of loose stools to obtain the final loose stool grade. Diarrhea index was obtained from the final loose stool grade multiplied with the loose stool rate.

As shown in Table 2, fecal scores of rats were recorded according to the Bristol stool scale to evaluate the fecal morphology [22]. According to the IBS-D classification criteria [23], loose stools (paste-like stools) or watery stools accounted for more than 25% of the total stool numbers, and hard or lumpy stools accounted for less 25% of the total stool numbers. The loose (paste-like) or watery stools and hard or lumpy stools corresponded to grades 1-2 and grades 6-7 of the Bristol classification, respectively.

### 2.3. Animal Grouping and Treatment

In our study, the rats were divided into a normal group for controls and a modeling group for further analysis. After successful establishment of the IBS-D model, rats in modeling group were randomly divided into four groups. As a result, we had five groups as follows:

 
*Group 1. Normal (NC group, n* *=* *8).* Rats took food and water freely.
 
*Group 2. IBS-D (MC group, n* *=* *8).* Rats took food and water freely.
 
*Group 3. IBS-D* *+* *Ginger-Partitioned Moxibustion (GPM Group, n* *=* *8).* Ginger slice (18 mm in diameter and 3 mm in thickness) was placed on the Tianshu acupoint (ST25), and a moxa cone (7 mm in diameter and 9 mm in height, weight 50 mg) was placed on the ginger slice and ignited. When a moxa cone burned out, it was replaced by a new one. The moxibustion intervention lasted for one week for 30 min/day. ST25 is located on a horizontal line 5 “cun” above the symphysis pubis and 2 “cun” lateral to the midline [24].
 
*Group 4. IBS-D* *+* *Mild Moxibustion (MM Group, n* *=* *8).* The ignited moxa stick (0.5 cm in diameter) was suspended approximately 1 cm above the Tianshu acupoint of rats for one week for 30 min/day. Ash deposited under the burning moxa stick was removed.
 
*Group 5. IBS-D* *+* *Laser Moxibustion (LM Group, n* *=* *8).* A laser system (STL808T1-4.0 W, Beijing Stone Laser Technology Co., Ltd.) with an output power of 125 mW, and a laser beam radius of 2.5 mm was applied on ST25 of rats for one week for 30 min/day.

Surface temperatures of all rats at the ST25 acupoint during moxibustion treatment were recorded. For the GPM group, temperature was recorded by T-type thermocouples (Omega Engineering Inc., Stamford, CT, USA), while, for the MM group and LM group, temperatures were recorded by infrared camera (FLIR^™^ T420). The details of temperature recording and analysis can be found in our previous report [16]. In addition, mental state, fur appearance, activity, and weight changes of rats were monitored during moxibustion intervention. Stools of rats were also analyzed as modeling process did.

### 2.4. Preparation of Acupoint and Colon Tissue Samples

After seven-day treatment, tissue samples from ST25 acupoint area (2 cm in length by 2 cm in width) and colon (3 cm in length) at 8 cm proximal to the anus were collected. Acupoint tissue samples were used to observe the expression of TRPV1 in the acupoint area. Each colon sample was divided into two pieces for separate histological and immunohistochemistry analysis. All tissue samples were fixed with 4% paraformaldehyde solution.

### 2.5. Histological Analysis

To detect the epithelial structure and infiltration of inflammatory cells, colon samples were stained with hematoxylin-eosin (H&E). The samples fixed in 4% paraformaldehyde solution were dehydrated, paraffin-embedded, and sectioned at a 4 *μ*m thickness. The colon tissue sections were soaked in hematoxylin staining solution for 5 min, followed by rinsing in running water several times, and placed into 1% hydrochloric acid alcohol for 2 s. Then, the sections were extracted and rinsed quickly with tap water. After that, the sections were placed into dilute lithium carbonate aqueous solution for about 30 s, followed by rinsing with tap water and dehydration with 80% ethanol, and finally, the sections were immersed in eosin staining solution for 20 s. The stained sections were placed into 95% ethanol (2 lanes, 10 s each), anhydrous ethanol (2 lanes, 2 min each), and xylene (2 lanes, 2 min each) in turn, and coverslips were mounted on the glass slides using neutral gum. All stained sections were observed with a light microscope.

### 2.6. Immunohistochemistry to Detect TRPV1 Expression

The expression of TRPV1 in the acupoint tissue and colonic tissue of all rats was detected by immunohistochemistry (IHC) staining. The paraffin sections of colon and acupoint tissue were deparaffinized in xylene I and II solutions for 10 min each and rehydrated in 100%, 95%, 85%, and 75% ethanol for 5 min. After washing with PBS for 30 min, tissue sections were immersed in 0.01 M citrate buffer (pH 6.0) and heated in a microwave at 98°C for 2.5 min, 1.5 min, and 1 min, with 15 min intervals for antigen retrieval. After cooling to room temperature, tissue sections were placed in 1% H_2_O_2_ for 10 min and washed in PBS. The tissue sections were incubated with endogenous peroxidase enzyme-blocking buffer for 10 min at room temperature, followed by washing in PBS. After 10 min of incubation with normal goat serum for blocking and washing in PBS, the sections were incubated with rabbit anti-TRPV1 antibody (bs-23927R, Bioss) overnight at 4°C. The sections were then rinsed with PBS and incubated with goat anti-rabbit/mouse IgG for 10 min at room temperature. After rinsing with PBS, the sections were incubated with streptavidin-HRP for 10 min at room temperature. Finally, DAB color development working solution was added on the tissue sections. The color development time was controlled by observation under the microscope, and when the best color development effect was achieved, the color development was discontinued by rinsing with tap water. After color development, the sections were restained with hematoxylin, dehydrated, and mounted with neutral gum. All stained sections were observed with a light microscope.

The positive expression fraction of TRPV1 was observed under the optical microscope as light yellow, brownish-yellow, or tan. Professional image analysis software Image-Pro Plus 6.0 was used for semiquantitative analysis of immunohistochemical-positive expression, and the mean optical density (MOD) was calculated. The tissue measurement area with staining tone on the image was selected randomly. Then, the integral optical density (IOD) and the size (or known as area of interest, AOI) of the selected area were calculated. The ratio of the integrated optical density to the positive area is the MOD; MOD = IOD/AOI. The MOD reflects the average depth of the positive signal, and a large MOD value is associated with strong protein expression, while a small MOD value is associated with weak protein expression.

### 2.7. Statistical Analysis

The results are presented as mean values ± standard error. Statistical analysis was performed using SPSS 21.0. One-way ANOVA was performed for experimental data among groups, and post-hoc tests were used for further analysis with significant effects. Significant differences were considered by a *P*-value of less than 0.05.

## 3. Results

### 3.1. Identification of IBS-D Model Rats

By comparing the appearance between the normal group and the modeling group, we found that rats in the normal group were active and alert. Their fur was white and shiny, and auricles were pale pink. The anus and tail were clean with no fecal staining. Rats in the modeling group appeared mentally fatigued with a thin body, were less active but aggressive, and liked to curl up in the corners. Their fur was withered and yellowish, and auricles were pale in color. Loose stools were stuck around their anus and tail.

Table 3 shows the statistics on food and water intake, weight change percent, and diarrhea index and Bristol stool scale of rats in the normal and modeling groups. After the modeling process, rats in the modeling group took less food and water compared with the normal group (*P* < 0.05). The weight change percent of rats in the modeling group was also much lower than that of the normal group (*P* < 0.05), and the weight change percent of IBS-D rats was negative, indicating that the weight of the rats was decreasing during the modeling process. After modeling, both the diarrhea index and Bristol stool scale of rats in modeling group were higher compared with the normal group (*P* < 0.05), and the Bristol stool scale classification for rats in the modeling group was in the range of diarrhea, as shown in Table 2. In all, our IBS-D model was successful.

### 3.2. Improvements in IBS-D Rats after Moxibustion Treatment

After one week of moxibustion treatment, rats in the three intervention groups showed improvement in their mental state. They increased self-motivated activities and their fur became shiny. No fecal material was found around the anus. Rats in the modeling group were thin and inactive. Fecal staining could be found around the anus and tail. The weight increases of rats in three intervention groups were higher than that in the modeling group, but no statistical differences in water and food intake were found among the groups.

**Figure 1:**
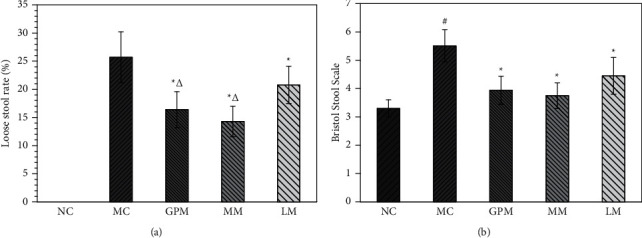
Comparison of rat stool among groups after treatment. NC: normal group; MC: IBS-D model group; GPM: IBS-D + ginger-partitioned moxibustion group; MM: IBS-D + mild moxibustion group; LM: IBS-D + laser moxibustion group. ^*∗*^Compared with MC, *P* < 0.05*;*^Δ^compared with LM, *P* < 0.05*;*^#^compared with NC, *P* < 0.05.

Figure 1 shows the comparison of rat stool among groups. We can see that moxibustion treatment improved diarrheal symptoms in IBS-D rats. Loose stool rate and Bristol stool scale of rats in three moxibustion treatment groups were lower than in the modeling group (*P* < 0.05). In addition, the loose stool rate and Bristol stool scale of rats in the GPM and MM groups were lower than in the LM group, but there were no statistical differences in the Bristol stool scale.

### 3.3. Histological Observation

**Figure 2:**
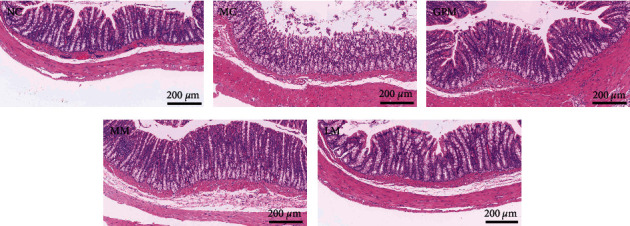
Pathological changes of colon tissue in rats (H&E staining, ×100). NC: normal group, MC: IBS-D model group; GPM: IBS-D + ginger-partitioned moxibustion group; MM: IBS-D + mild moxibustion group; LM: IBS-D + laser moxibustion group.

Figure 2 shows the results of hematoxylin-eosin (H&E) staining of rat colon tissue under a light microscope x100. There were no obvious pathological changes in the H&E staining of rat colon tissue in each group, suggesting that IBS-D is a nonorganic gastrointestinal disease. The colon tissue and mucosal structure of rats in the normal group and each intervention group were intact without hyperplasia of fibrous connective tissue. The colonic mucosa of rats in the IBS-D model group showed low-grade inflammation, cellulose exudation, and submucosal vasodilation but no obvious erosion and ulcer formation.

### 3.4. TRPV1 Expression in the ST25 Acupoint Area

**Figure 3:**
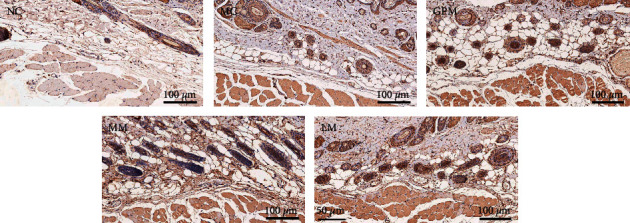
TRPV1 expression of acupoint tissue in rats (IHC staining, ×200). NC: normal group; MC: model group; GPM: IBS-D + ginger-partitioned moxibustion group; MM: IBS-D + mild moxibustion group; LM: IBS-D + laser moxibustion group.

**Figure 4:**
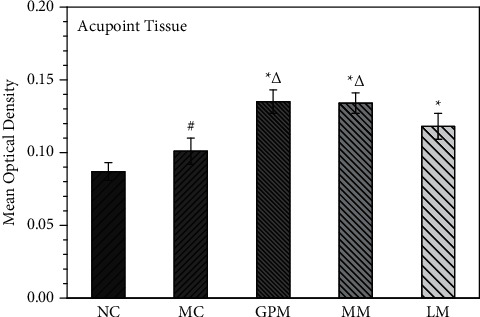
TRPV1 expression of acupoint tissue in rats. NC: normal group; MC: model group; GPM: IBS-D + ginger-partitioned moxibustion group; MM: IBS-D + mild moxibustion group; LM: IBS-D + laser moxibustion group. ^#^Compared with NC, *P* < 0.05*;*^*∗*^compared with MC, *P* < 0.05*;*^Δ^compared with LM, *P* < 0.05.

Figure 3 shows the IHC staining of the TRPV1 receptor in acupoint tissues of rats (×200). The positive expression of TRPV1 receptor appears brownish-yellow or tan.  Figure 4 shows the comparison of the MOD values of TRPV1 receptor expression in acupoint tissues of rats after moxibustion intervention (IHC staining, × 200). The MOD values of TRPV1 in the acupoint tissues of rats in the model group were higher than those in the normal group, and the difference was statistically significant (*P* < 0.05), indicating that TRPV1 receptor expression in the acupoint tissues of IBS-D rats was increased under pathological conditions. The expression of TRPV1 in the acupoint tissues of rats in the three moxibustion intervention groups was higher than that in the model group (*P* < 0.05), indicating that TRPV1 receptor expression was further increased after moxibustion intervention.

The expression of TRPV1 receptors in the acupoint tissues of GPM and MM were higher than that of LM (*P* < 0.05), indicating that GPM and MM were more effective than LM in increasing TRPV1 expression in the acupoint tissues. The effect on TRPV1 expression between GPM and MM showed nearly no difference.

### 3.5. TRPV1 Expression in the Colon Tissue

**Figure 5:**
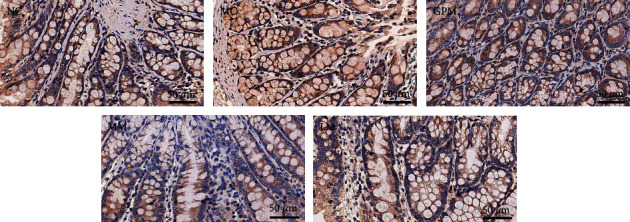
TRPV1 expression of colon tissue in rats (IHC staining, ×400). NC: normal group; MC: model group; GPM: IBS-D + ginger-partitioned moxibustion group; MM: IBS-D + mild moxibustion group; LM: IBS-D + laser moxibustion group.

**Figure 6:**
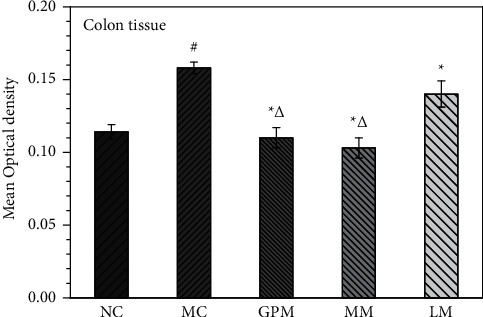
TRPV1 expression in rat colon tissue. NC: normal group; MC: model group; GPM: IBS-D + ginger-partitioned moxibustion group; MM: IBS-D+ mild moxibustion group; LM: IBS-D + laser moxibustion group. ^#^Compared with NC, *P* < 0.05*;*^*∗*^compared with MC, *P* < 0.05; ^Δ^compared with LM, *P* < 0.05.

Figure 5 shows the IHC staining of the TRPV1 receptor in colon tissues of rats (×400). The positive expression of the TRPV1 receptor appears brownish-yellow or tan.  Figure 6 shows the comparison of the MOD values of TRPV1 receptor expression in colon tissues of rats after moxibustion intervention (IHC staining, ×400). The mean optical density values of TRPV1 in the colon tissues of rats in the model group were higher than those in the normal group, and the difference was statistically significant (*P* < 0.05), indicating that the modeling process in IBS-D rats could induce the expression of the.

The expression of TRPV1 in the colon tissues of rats in the three moxibustion intervention groups was lower than that in the model group (*P* < 0.05), indicating that moxibustion interventions effectively decreased the expression of TRPV1 receptor. By comparing the expression of TRPV1 receptors among three moxibustion treatment groups, it was the lowest in GPM, followed by MM, and highest in LM, but no significant statistical differences were observed between GPM and MM.

### 3.6. Temperature Profiles of the Acupoint during Moxibustion Intervention

**Figure 7:**
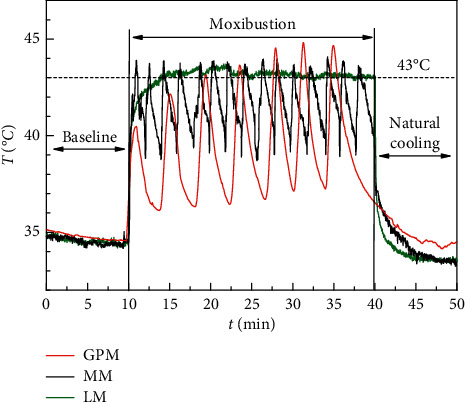
Temperature of rats in ST25 acupoints during moxibustion. GPM: ginger-partitioned moxibustion; MM: mild moxibustion; LM: laser moxibustion.

Figure 7 shows temperature profiles of rat ST25 acupoints during moxibustion intervention. Ginger-partitioned moxibustion group (GPM), mild moxibustion group (MM), and laser moxibustion group (LM) are represented by a red line, black line, and green line, respectively. The temperature curve of GPM presents a wave-like change that first rises and then falls, with a total of seven wave crests. The values of its first two crests were around 40.5°C and 42°C, respectively. After the third crest, the highest temperature during GPM reached approximately 43°C. The temperature curve of MM also showed a wave-like change but with a faster frequency compared with GPM. The value of the crest during MM also reached approximately 43°C. For LM, rat temperature increased quickly to around 40 upon the initiation of the laser system and reached approximately 43°C within next 3 min. Then, the temperature remained fairly stable at 43°C until the end of laser treatment.

## 4. Discussion

### 4.1. The Therapeutic Effect of Moxibustion Therapies on IBS-D Rats

According to the clinical manifestations of IBS-D, Chinese medicine classifies it as diarrhea and abdominal pain. The *JingYue QuanShu Xiexie* says “the origin of diarrhea is always due to the spleen and stomach,” pointing to internal injury to the spleen and stomach as the root cause of diarrhea. According to the concept of seeking the root cause of the disease in Chinese medicine, spleen deficiency is the root of the pathogenesis of IBS-D, and its pathogenesis is related to the dysfunction of the spleen, liver, kidney, and other internal organs. Although the etiology of diarrhea is complex, the basic pathological changes are spleen deficiency and dampness obstruction. Meanwhile, the liver and spleen are physiologically coordinated with each other, while pathologically they affect each other, with the liver being responsible for drainage and the spleen being responsible for transportation and transformation. If the qi in liver is stagnant and crosses to the spleen, it leads to spleen dysfunction, spleen deficiency, and liver exuberance. Then, abdominal pain and diarrhea occur as a result.

The *Yixuerumen Zhenjiu* says “the deficient patients get moxibustion, so that the fire is helpful for their yuan yang; the sufficient patients get moxibustion, so that their sufficient pathogenic factors disperse with the fire; the cold patients get moxibustion, so that their qi will be rewarmed.” Moxibustion plays the role of “warm dredging” and “warm nourishing” via thermally stimulating the body. The “moxibustion can make a supplement for the spleen and stomach” is described in the *Weishengbaojian*, which also means that moxibustion can warm the yang of the spleen and stop diarrhea. In Traditional Chinese Medicine, ST25 is a “mu” point of the large intestine, which regulates the function of the large intestine, spleen, and stomach [25]. According to our previous investigation on the selection rules of acupoint on treating IBS-D, ST25 is the most used acupoint during acupuncture and moxibustion intervention [26]. The results in this study indicated that all three moxibustion interventions on ST25 can improve the symptoms in IBS-D rats, showing a good therapeutic effect. This was shown by a significant improvement in the general condition of the rats and decreases in loose stools, the diarrhea index, and Bristol stool scale and increase of normal stools after treatment. In addition, GPM and MM showed a better therapeutic effect compared with LM, but no significant difference was observed between GPM and MM.

### 4.2. The Role of TRPV1 in IBS-D Management by Moxibustion Therapies

TRPV1 is one of the ligand-gated, nonselective positive ion channels that is activated when it binds to the corresponding ligand, and the channel opens to allow inward flow of Ca^2+^, inducing biological effects [27]. Activation of TRPV1 induces the release of neuropeptides, such as substance P and calcitonin-related peptides, from neurons and their fibers [28]. TRPV1 acts as an injury receptor that responds to heat and a variety of chemicals, such as capsaicin, noxious thermal stimuli (>43°C), voltage, and acidic pH [18], so it plays an important role in pain physiology and pharmacology. Some studies have confirmed that moxibustion thermal stimulation can effectively regulate the expression of TRPV1, and TRPV1 is a key target in producing the therapeutic effects of moxibustion [19]. Comparison of the effects of moxibustion-like warm stimulation at different temperatures on gastric motility in TRPV1 knockout (TRPV1-/-) mice and C57BL/6 wild-type mice showed that moxibustion-like stimulation above 42°C modulated gastric motility significantly, while TRPV1 knockout significantly reduced the related effects [29].

TRPV1 is widely expressed within sensory neurons innervating the gastrointestinal tract and its fibers and can be involved in various functions, such as protection of the gastrointestinal mucosa, gastrointestinal motility, sensation, and endocrine activity, and has important significance in the pathogenesis of visceral hypersensitivity and gastrointestinal motility disorders in functional gastrointestinal diseases [30–32]. The hypersensitivity of the intestine to pain sensation is one of the main pathological features of IBS, and the main mechanism of its formation is closely related to the increased sensitivity of intestinal receptors and abnormal excitation of sensory afferent nerves. It has been found that activation of TRPV1 in the gastrointestinal tract opens Ca^2+^ channels and causes the release of neurotransmitters such as vasoactive intestinal peptide and SP from neurons and their fibers, thereby regulating visceral sensation and gastrointestinal dynamics and triggering or facilitating the pain process [33]. Clinical trials found that the expression level of TRPV1 in the colonic mucosa of IBS patients was 3.5 times higher than that in healthy control subjects and was positively correlated with the degree of abdominal pain [34–36]. The sensitivity of rats with TRPV1 knockout was significantly reduced when their gastrointestinal tract was mechanically stimulated [37]. Thus, TRPV1 plays an important role in the pathogenesis of IBS-D and in the generation of visceral hypersensitivity [38, 39].

In this study, we found that the expression of TRPV1 receptors in both acupoint tissues and colonic tissues of IBS-D model rats was higher than that in normal healthy rats, indicating that the acupoint area may become activated from a dormant state, and the colonic tissues showed a pathologically activated state. The intervention of three moxibustion treatments further increased the expression of the TRPV1 receptor in acupoint tissue while decreasing the expression of the TRPV1 receptor in colon tissue. Moxibustion might play a role in the treatment of IBS-D by thermal stimulation to activate TRPV1 channels in the acupoint tissue and induce acupoint functions, which in turn inhibit the pathological activation state of TRPV1 ion channels in the colon, followed by a reduction of intestinal hypersensitivity and regulation of colon motility, as well as improvements in abdominal pain and diarrheal symptoms.

The investigation on the signaling pathway between acupoint function and the reaction of viscera is a significant and crucial topic for acupoint intervention on intestinal diseases. Some innovative efforts have been made to reveal the possible signaling pathway of acupoint intervention on IBS-D. For example, Chen et al. [40] and Li [41] proposed that acupoint intervention might modulate Pirt-TRPV1 signaling pathway and PLC-TRPV1 signaling pathway, respectively, and inhibit the abnormal expression of TRPV1 in colonic tissues, followed by the alleviation of visceral pain. However, the exact pathogenesis of IBS remains unclear, as it has become apparent that multiple pathways are activated in the condition, including inflammation, immunology, neurology, and psychology [42]. More biomedical experiments are needed in the future to explore how acupoint inhibits the pathological activation of TRPV1 ion channel in colon in the treatment of IBS-D by acupoint intervention.

### 4.3. Dynamic and Repeated Thermal Stimulation Induce Sensitization of Receptors and Acupoints

The activation of TRPV1 receptors is closely related to the temperature of the thermal stimulus. It was shown that a rapid temperature rise enables relatively fast activation of TRPV1 receptors, with channel currents reaching a plateau in less than 500 ms [43]. In addition, the temperature threshold at which TRPV1 is activated is not fixed but regulated by chemical ligands and channel phosphorylation status. For example, when the TRPV1 receptor is phosphorylated by protein kinase C, its channel can be opened at the normal body temperature [44]. Studies have shown that, after the initial burning sensation following local skin application of capsaicin, sensory nerves become desensitized to heat, capsaicin, and other types of stimuli, and removal of extracellular calcium ions can greatly diminish or even eliminate the desensitization phenomenon [45]. When high concentrations of capsaicin are applied to sensory nerve cells over a long period of time, TRPV1 channel currents show an initial spike followed by a low flat “plateau” in a biphasic manner [46]. Sustained thermal stimulation can also lead to desensitization of TRPV1 receptors in a noncalcium-dependent manner [47]. When TRPV1 receptor desensitization occurs, the probability of receptor activation or the value of channel currents in response to noxious thermal stimulation will decrease.

Research from multiple disciplines supports the notion that the temporal characteristics of repetitive drug, electrophysiological, or psychological stimuli influence the direction of cellular and behavioral adaptation. Chronic continuous stimulation is usually associated with the development of tolerance, whereas intermittent stimulation may have the opposite effect and be associated with sensitization or reverse tolerance [48]. Studies have shown that repetitive stimulation can cause sensitization of the nervous system, or an enhancement of the response, generally arising from injury to the receptors [49]. For example, Bessou and Perl [50] found that repetitive noxious thermal stimulation (>45°C) can induce the sensitization of cutaneous C receptor. In recent years, the tolerance and sensitization of acupoints have gradually become a focus of traditional Chinese medicine research [51–53]. The study of acupoint analgesia by electroacupuncture found that the analgesic effect of electroacupuncture with a frequency of once a day was not cumulative but weakened, with the increase in the intensity of electroacupuncture resulting from the emergence of acupoint tolerance [54]. Heat-sensitive moxibustion usually showed a greater therapeutic effect compared with traditional moxibustion [55, 56]. Moreover, sensitized acupoint stimulation could improve gastrointestinal function in irritable bowel syndrome rats, either through electroacupuncture or moxibustion [57, 58].

Based on the previously mentioned analysis, different moxibustion therapies could induce different temperature curves on the skin of rat acupoints. The fluctuating temperature profiles of GPM and MM could provide dynamic and repetitive thermal stimulation on the acupoint area and perhaps induce acupoint sensitization for better efficacies. The temperature profile of LM basically remained stable after a rapid increase, which might lead to the desensitization of TRPV1 receptors and tolerance in the acupoint, reducing the therapeutic effect as a result. Therefore, for the clinical application of moxibustion therapies, it is recommended to perform dynamic and repetitive thermal stimulation on the acupoints to induce acupoint sensitization and thus improve the therapeutic effect of moxibustion.

## 5. Conclusion

In summary, we compared the therapeutic effects of pretreatment of the Tianshu (ST25) acupoint on IBS-D model rats under three moxibustion therapies. Some conclusions are as follows: GPM, MM, and LM with temperature of 43°C could effectively improve the symptoms of IBS-D rats. Moxibustion might play a role in the treatment of IBS-D by activating TRPV1 channels in the acupoint tissue and induce acupoint functions, which in turn inhibit the pathological activation state of TRPV1 ion channels in the colon, followed by improvements in abdominal pain and diarrheal symptoms. LM with stable temperature might lead to the desensitization of TRPV1 receptors and tolerance in the acupoint. The fluctuating temperature profiles of GPM and MM could provide dynamic and repetitive thermal stimulation for the acupoint area and perhaps induce acupoint sensitization for better efficacy. Therefore, dynamic and repetitive thermal stimulation is recommended in the application of moxibustion.

## Figures and Tables

**Table 1:** Grades corresponding to the pollution diameter of loose stool.

Pollution diameter	Grade
<1.0 cm	1
≥1.0 cm and <2.0 cm	2
≥2.0 cm and <3.0 cm	3
≥3.0 cm	4

**Table 2:** Bristol stool scale classification of rat stool samples.

Performance	Characteristics	Score
Constipation	Separated hard lumps	1
	Sausage-like lumps	2

Normal	Strip-shaped excrement with fractures	3
	Soft and slick snake-like strip	4

Diarrhea	Soft crumbs with clear boundary	5
	Fluffy paste with ragged borders	6
	Watery stool without solid lumps	7

**Table 3:** Comparison of food and water intake, weight change rate, and stool of rats between normal and modeling groups after modeling.

Groups	*n*	Food intake	Water intake	Weight change percent	Diarrhea index	Bristol stool scale
Normal	8	24.4 ± 2.2	39.2 ± 4.2	26.9 ± 2.8	0	3.2 ± 0.4
Modeling	32	4.4 ± 0.5^*∗*^	25.0 ± 3.5^*∗*^	−17.4 ± 8.4^*∗*^	0.7 ± 0.2^*∗*^	5.7 ± 0.8^*∗*^

^*∗*^Compared with the normal group, *P* < 0.05.

## Data Availability

The data used to support the findings of this study are available from the corresponding author upon request.
